# Large-Scale Screening for Targeted Knockouts in the *Caenorhabditis elegans* Genome

**DOI:** 10.1534/g3.112.003830

**Published:** 2012-11-01

**Authors:** The C. elegans Deletion Mutant Consortium

**Affiliations:** * Molecular and Cell Biology, Oklahoma Medical Research Foundation, Oklahoma City, Oklahoma, 73104; † Department of Physiology, Tokyo Women’s Medical University School of Medicine, Tokyo 162-8666, Japan; ‡ Department of Zoology and Michael Smith Laboratories, University of British Columbia, Vancouver, British Columbia, V6T 1Z3 Canada

**Keywords:** genomics, knockouts, deletion mutations, multi-gene families

## Abstract

The nematode *Caenorhabditis elegans* is a powerful model system to study contemporary biological problems. This system would be even more useful if we had mutations in all the genes of this multicellular metazoan. The combined efforts of the *C. elegans* Deletion Mutant Consortium and individuals within the worm community are moving us ever closer to this goal. At present, of the 20,377 protein-coding genes in this organism, 6764 genes with associated molecular lesions are either deletions or null mutations (WormBase WS220). Our three laboratories have contributed the majority of mutated genes, 6841 mutations in 6013 genes. The principal method we used to detect deletion mutations in the nematode utilizes polymerase chain reaction (PCR). More recently, we have used array comparative genome hybridization (aCGH) to detect deletions across the entire coding part of the genome and massively parallel short-read sequencing to identify nonsense, splicing, and missense defects in open reading frames. As deletion strains can be frozen and then thawed when needed, these strains will be an enduring community resource. Our combined molecular screening strategies have improved the overall throughput of our gene-knockout facilities and have broadened the types of mutations that we and others can identify. These multiple strategies should enable us to eventually identify a mutation in every gene in this multicellular organism. This knowledge will usher in a new age of metazoan genetics in which the contribution to any biological process can be assessed for all genes.

It has been fourteen years since the *C. elegans* Sequencing Consortium published the landmark paper on the sequence of the genome of this small roundworm (*C. elegans* Sequencing Consortium 1998). *C. elegans* was the first metazoan to reach this historic milestone, and as such, it offered the unprecedented opportunity for a comprehensive genetic and molecular dissection of the development and physiology of different cell types and tissues and of the genesis of organs. In the intervening years, studies on this nematode have not disappointed, largely because of the accumulated availability of thousands of mutations throughout the genome.

Although it was clear to many in 1998 that to fully exploit the potential of this model system we would need to obtain mutations in all the genes, the way forward was not immediately obvious. This was because, unlike yeast, there was no homologous recombination system available to quickly generate targeted gene disruptions. Fourteen years later, the yeast *Saccharomyces cerevisiae* is still the only eukaryote with a set of deletions for all genes in the genome (Winzeler *et al.* 1999; Giaever *et al.* 2002).

In May 1998, a meeting was convened at the Sanger Center in Hinxton, England, to discuss the feasibility of mounting a large-scale *Caenorhabditis elegans* project to mutate every single gene in this organism. Many of those present were working on strategies to obtain deletions in individual genes, and the relative merits and cost of each strategy was discussed. What emerged from the meeting was the formation of a consortium of laboratories dedicated to providing the research community with gene knockouts (*i.e.* deletions or KOs) upon request [reviewed in Moerman and Barstead (2008)]. Initiatives similar in concept and ultimate goals to the *C. elegans* deletion project are underway for several model organisms. The international Knockout Mouse Consortium (http://www.knockoutmouse.org/) and the Zebrafish Mutation Project (http://www.sanger.ac.uk/Projects/D_rerio/zmp/) are two excellent examples.

The *C. elegans* Deletion Mutant Consortium as it stands today consists of three laboratories: the laboratory of Robert Barstead, located at the Oklahoma Medical Research Foundation (OMRF) in Oklahoma; the laboratory of Shohei Mitani, located at Tokyo Women’s Medical University, Tokyo, Japan; and the laboratory of Donald Moerman, located at the University of British Columbia (UBC) in Vancouver, Canada. While differing in some details, the fundamental screening strategy for deletions by each consortium lab is the same: populations of animals are exposed to a mutagen, and polymerase chain reaction (PCR) DNA amplification is used to identify deletions at target loci (Figure 1). Through a sib selection process over several generations, a single animal is eventually isolated harboring the deletion. Standard protocols for PCR/deletion screening are described in detail elsewhere (Zwaal *et al.* 1993; Jansen *et al.* 1997; Liu *et al.* 1999; Gengyo-Ando and Mitani 2000; Barstead and Moerman 2006; also see http://www.zoology.ubc.ca/%7Edgmweb/research1_pcr.htm and the summary of this article). More recently, we have added comparative genome hybridization and massively parallel short-read sequencing technologies (whole-genome sequencing) to obtain gene mutations (Maydan *et al.* 2007, 2010; Flibotte *et al.* 2010). However, for this report, the vast majority of mutations generated are the result of PCR/deletion screening.

**Figure 1:**
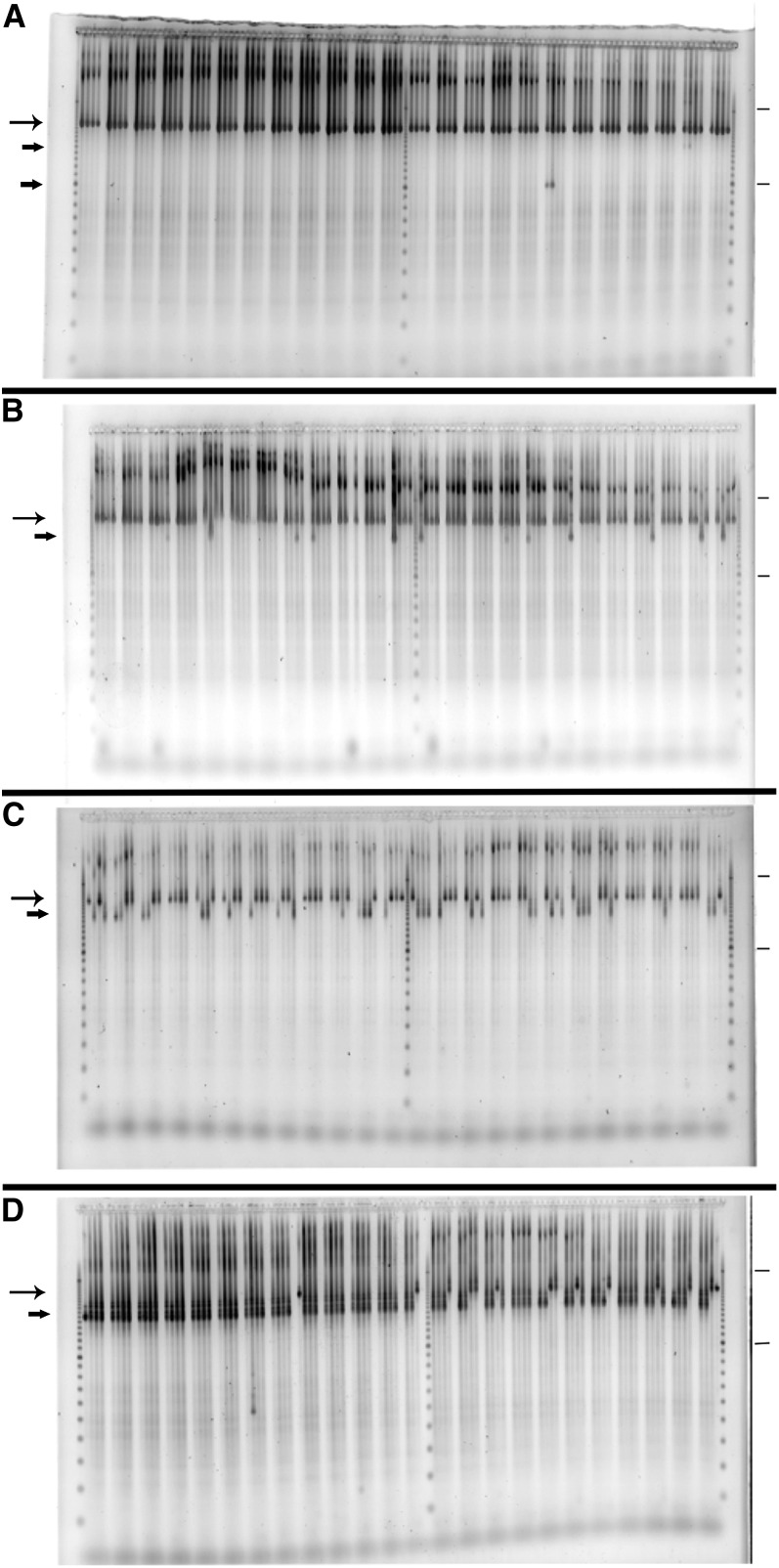
PCR/deletion screening. Worms were grown in liquid culture in arrays of 96. A portion of each well was harvested for DNA preparation while the rest was frozen for later retrieval of animals. DNA was pooled into a grid of rows and columns, which could later be used to address individual wells. Nested PCR primers were designed for amplification of target region. Primers were designed using the program Aceprimer (McKay and Jones 2002). Shown are four slab gels stained with SYBR Gold used to identify deletions (faster running bands) from the wild-type DNA (slower bands). The four panels are (A) the initial screen, (B) first sib, (C) second sib, and (D) third sib. The WT band for this primer set is 2099 bp (long arrow), and the marker is a 100-bp molecular weight ladder with strong intensity bands at 1 kb and 3 kb (marked by horizontal bars on right hand side of image). The screening image (A) shows two different hits (in duplicate screening, short arrows), one at just over 1 kb (18^th^ set of four lanes, in first two lanes of the four), and one at about 1.6 kb (23^rd^ set of four lanes, in first two lanes). The one at 1 kb passed addressing but was not recovered in sib selection, so the remaining images (B–D) are for the 1.6 kb candidate. Through rounds of sib selection, one enriches for animals segregating the deletion band. This enrichment proceeds from initial detection in (A), a mix of hundreds to thousands of animals, to first-round sibbing in (B), tens of animals, and finally single animal picks in sib2 (C) and sib3 (D), where one has single animals segregating the deletion band. For sib3 (D), there are 4 × 24 single-worm populations, 1–4. Populations 1 and 2 are in the first half of the comb, and 3 and 4 are in the second half. Set 3 is actually 24/24 positive, whereas the others are less than that, so set 3 was picked to go forward as it was homozygous at that point. The example shown is gk3287 in the gene F11E6.1 (gba-3).

## Materials and Methods

### Nematode culture, mutagenesis, and DNA preparation

Nematodes were generally grown as previously described (Brenner 1974), with various modifications to suit the needs of our individual laboratories. All mutagenesis was carried out on the canonical wild-type strain N2, with each group using its own subculture of the strain. Mutagens employed include ethyl methanesulfonate (EMS), formaldehyde, and trimethyl psoralen treatment followed by UV irradiation (UV/TMP), generally under standard protocols. Modifications included variation of EMS dose; variation of TMP concentration and the length of UV irradiation; preparation of TMP concentrate in acetone or DMSO; and use of various types of equipment to deliver UV at 340 uW/cm2. Genomic DNA preparation for PCR deletion screens was usually as crude Proteinase K lysates of samples from library populations. DNA preparation for other procedures was performed using the PureGene Genomic DNA Tissue Kit (Qiagen catalog number 158622), following a supplementary Qiagen protocol for nematodes.

### Deletion discovery by polymerase chain reaction (PCR)

Our three laboratories followed a basic protocol that underwent various types of development, fine-tuning, and specialization throughout the period of its application. In its simplest form, the protocol involves (1) design and synthesis of nested primer sets to drive detection of deletions in a large set of interesting target genes; (2) generation of a worm library representing anywhere from 125,000 to 1.4 million mutagenized genomes; (3) sampling of the library to yield enough DNA for wide screening, while preserving enough of the original populations that recovery of mutant animals was not compromised; (4) preparation of population DNA samples by crude Proteinase K lysis; (5) pooling of population DNAs to reduce the number of PCRs necessary to screen the entire library for deletions; (6) screening by nested PCR and agarose gel analysis to identify pools containing deletion PCR products (nested PCR provides both high sensitivity in complex pools and high specificity); (7) population addressing PCR and gel analysis to identify a single population conaining each particular deletion detected in pools; (8) recovery of surviving worms from individual library populations; (9) recovery of single animals heterozygous for each deletion through a stepwise program of sibling selection (several rounds of expansion by regrowth, sampling, DNA preparation, PCR, and gel analysis at progressively lower initial seed density until single-parent deletion populations were identified); (10) creation of stable deletion lines by establishment of homozygosity or construction of genetically balanced recessive lethal deletion strains; and (11) elucidation of deletion breakpoints by Sanger sequencing of PCR deletion products.

Various alterations to this protocol were made by our individual laboratories in several areas, including mutagenesis methods and agents, library complexity, use of frozen or live libraries, use of the poison primer PCR method (Edgley *et al.* 2002), and development of robotic solutions for various processing steps. Details for some of these variations can be found in published work (Gengyo-Ando and Mitani 2000; Barstead and Moerman 2006) or on the Moerman lab website (http://www.zoology.ubc.ca/~dgmweb/research1.htm).

### Deletion discovery by comparative genome hybridization and whole-genome sequencing

Comparative genome hybridzation (CGH) allows copy number interrogation of an entire mutant genome in a single experiment. For this work, we applied the method to several different types of nematode strains to identify new deletions: wild *C. elegans* isolates (Maydan *et al.* 2007, 2010); balanced lethals isolated after mutagenesis (Maydan *et al.* 2007; Edgley *et al.* 1995); unmarked lines resulting from mutagenesis and clonal propagation (“anti-twitchers”); and homozygous deletion lines resulting from standard PCR screening (primarily *gk* alleles identified in the Moerman lab).

CGH protocols generally followed those of Maydan *et al.* (2007), except that processing steps for nearly all experiments were performed in-house instead of at Roche NimbleGen. For whole-genome sequencing (WGS), we followed the protocol previously described (Flibotte *et al.* 2010). “Anti-twitcher” strains for both CGH and WGS were generated and analyzed using the basic protocol for isolation of *unc-22* strains, except that F2 non-twitchers were picked in 1% nicotine and these were propagated clonally through the F10 generation. (Isolation of F1 heterozygous *unc-22* animals, called twitchers because they vibrate in 1% nicotine solution, ensured that resulting lines were adequately mutagenized, and selection of non-*unc-22* animals at F2, the anti-twitcher screen, produced lines without obvious morphological phenotypes.) Homozygous viable *gk* deletion strains isolated from standard PCR screening (protocols listed on the Moerman lab website; http://www.zoology.ubc.ca/~dgmweb/research1.htm) were analyzed by CGH both for validation of the deletion isolated by PCR (see below) and to determine whether extra deletions unrelated to the PCR screening target were present in the genome.

### Elucidation of deletion breakpoints

Deletion breakpoints were determined by Sanger sequencing of deletion PCR products and analysis by BLAST against the *C. elegans* genome. PCR products from deletion-positive reactions were pooled and purified using standard PCR-cleanup spin columns (for example, the Qiagen Qiaquick PCR Purification Kit, catalog number 28106) and subjected to Sanger sequencing from both ends using the left and right internal primers from the nested set used for isolation. Note that unoptimized nested PCR typically yields only the shorter deletion product from reactions on heterozygotes, so it was not necessary to obtain pure homozygous samples to get good quality sequence. The sequence data were analyzed with standard nucleotide BLAST (for example, using the BLAST server at www.ncbi.nlm.nih.gov), and deletions were identified as discontinuities in the matches between query and subject in the correct genomic region (that is, between the PCR primers used for isolation) and of a size consistent with the observed band shift on agarose gels.

Deletion breakpoints were found to be of three basic types: clean breaks, breaks with one or more bases that could be assigned to either side of the breakpoint (“ambiguous” breaks), and breaks with one or more bases of inserted material (“insertion” breaks). Graphical display of breakpoints in WormBase requires a discrete pair of flanking sequences for each deletion, so we developed a standard for reporting ambiguous and insertion breaks. For ambiguous breaks, we calculated the left breakpoint at the rightmost possible position; for insertion breaks, we calculated breaks to maximize the left and right matching portions within the amplicon and to minimize the insertion size.

### Deletion validation

Some deletions isolated by the PCR method were discovered to be non-mutant (various investigator reports, data not shown), and in at least some cases, it was shown that under certain conditions a wild-type PCR product from flanking primers could be generated. We undertook a program of deletion validation to improve the overall quality of the materials generated by our projects.

The initial method for this validation was a diagnostic PCR, in which homozygous viable deletion strains were subjected to PCR with flanking primers to confirm the presence of the deletion, and a PCR on deletion and wild-type templates with one primer internal to the deletion and one external (the “diagnostic” pair - a product of predicted size should result from the wild-type template but not from the deletion template). Presence of a predicted product from a deletion template was taken to indicate that the wild-type gene was still present in the genome and presumably functional. Deletion strains failing this test were discarded.

The PCR diagnostic was abandoned in favor of CGH validation when we began to analyze PCR deletion strains for extra deletions. In this CGH diagnostic, both homozygous and balanced heterozygous deletions could be validated if CGH probes existed on our standard array design, as single-copy and two-copy losses are readily detectable. For this assay standard, CGH protocols were employed, but the data were analyzed both for new deletions and for confirmation that the deletion detected by PCR was missing by CGH criteria.

### Strain and data distribution

Strains produced by our three laboratories are made available to the research community in two different ways. The Mitani lab handles primary strain distribution itself, subject to a Materials Transfer Agreement, and it distributes both homozygous and unstabilized heterozygous lines. The Barstead and Moerman labs rely on the Caenorhabditis Genetics Center (CGC) for primary strain distribution, which requires that strains be stabilized. To accommodate stabilzation, a system was developed in which both laboratories did primary PCR screening and isolated homozygous viable lines, but persistent heterozygotes were shipped to the Moerman lab for stabilization by further sib-selection, leading to homozygous lines or genetic balancing of true lethal mutants. As a result, the Barstead lab contributed homozygous *ok* deletion strains directly to the CGC, and the Moerman lab contributed to the CGC all strains stabilized in Vancouver, whether they carried *gk* or *ok* deletion alleles. For strains submitted to the CGC, we pioneered shipment of frozen strains on dry ice to minimize handling steps and increase the number of strains that could be sent at one time.

Deletion breakpoint data were generated primarily in the Mitani lab (for *tm* alleles) and the Moerman lab (for *gk* and *ok* alleles). We worked closely with staff at WormBase to develop a graphical display of deletion extents in the genome browser, and to streamline data submission protocols to minimize the time between submission and appearance. For strains submitted to the CGC, complete database entries were prepared in the format of their in-house system to speed incorporation in their on-line strain list and thus get materials into the research community faster.

## Results and Discussion

### Targeting knockouts is largely driven by user requests

As it was clear early in the project that our efforts would be labor intensive, we did not want to spend valuable resources obtaining mutations in genes that would not be utilized by the research community. Therefore, we decided that our search for gene deletions would be motivated primarily by requests from *C. elegans* researchers. The wisdom of this decision can be seen in the approximately 1500 publications that utilized alleles generated by our group. Until recently, all requests were handled through two websites, one at the OMRF in Oklahoma and one in Tokyo, Japan. Going forward, all requests should be submitted through the website in Japan at http://shigen.lab.nig.ac.jp/c.elegans/index.jsp. The only priority for screening is date of submission. Genes are screened repeatedly against new mutation libraries, with different primer sets if necessary, until a deletion is obtained. The only exception to the request guideline is that we, after discussion with our Scientific Advisory Board and the community, are making a concerted effort to obtain mutations in all 941 transcription factors and 416 kinases (Reece-Hoyes *et al.* 2005; Weirauch and Hughes 2011; Manning 2005).

A survey of the 20,377 protein-coding genes in WormBase (WS220) reveals 6764 genes with associated molecular lesions that are either deletions or nonsense mutations. Our laboratories are responsible for 6841 mutations in 6013 genes. To place this number in perspective, in 1998 there were fewer than 500 genes with associated molecular lesions. The bulk of the mutations identified by our group are deletions identified after PCR screening for requested genes. There are also several deletions identified by CGH screening of mutagenized animals for either viable or lethal deletions (Maydan *et al.* 2007). We have included single-gene and multi-gene deletions in large multi-gene families from CGH screens of wild strains of *C. elegans* (Maydan *et al.* 2007, 2010; denoted as *niDf*, natural isolate deletions in WormBase). We have also included nonsense mutations and splicing defects derived from our WGS pilot project in the current report (Flibotte *et al.* 2010). Note, however, that we have not included missense mutations or any resequencing data beyond curated WormBase WS220 genes. Our calculation of 6841 mutations in 6013 genes is exclusive of about 500 small deletions we have identified that are limited to introns. If a deletion does not extend across at least one exon boundary, we consider it a silent allele, and it is not included in our estimate of mutated genes.

### Quality control and strain and data archiving

Once a mutation is identified and a homozygous or persistent heterozygous strain is established, quality control (QC) occurs at a number of levels. All deletion mutations are sequenced. As the mutagens employed can cause double-strand breaks in the DNA, the mutations we identify through PCR are most commonly generated through non-homologous end joining during DNA repair. Consequently, we observe a range in deletion sizes as well as deletions accompanied by duplications of flanking sequences and even insertions of DNA from elsewhere in the genome. Of 4101 *gk* and *ok* deletions, 1097 are accompanied by additional duplicated or insertion sequences. This additional DNA is often only a single or at most a few bases, but occasionally can be as large as a 2 kb insert. There are 87 cases of inserts over 100 bases in length among the 1097 deletions with accompanying insertions. Although there is a range in size of the deletions, they are all less than 3 kb due to the placement of the flanking PCR primers. Occasionally, we generate strains containing both an intact and deleted target gene. Based on reports from others and our own experimental data where we have tested 183 strains by array CGH, this happens in less than 1% of mutant strains.

A bonus of using CGH as a QC step on deletion strains isolated after PCR screening is that we often find additional deletions in the same strain. For example, in the set of 183 CGH validation strains, we found 57 additional gene deletions, including 1 strain with 5 deletions in total. This result with CGH analysis and our recent finding of over 300 mutations in strains after standard mutagenesis procedures using WGS (Flibotte *et al.* 2010) should serve as a warning to those using the strains that the strains need to be outcrossed extensively to remove extraneous mutations. We urge and indeed rely on the nematode user community to remove extraneous background mutations before a phenotypic characterization of these strains.

**Figure 2:**
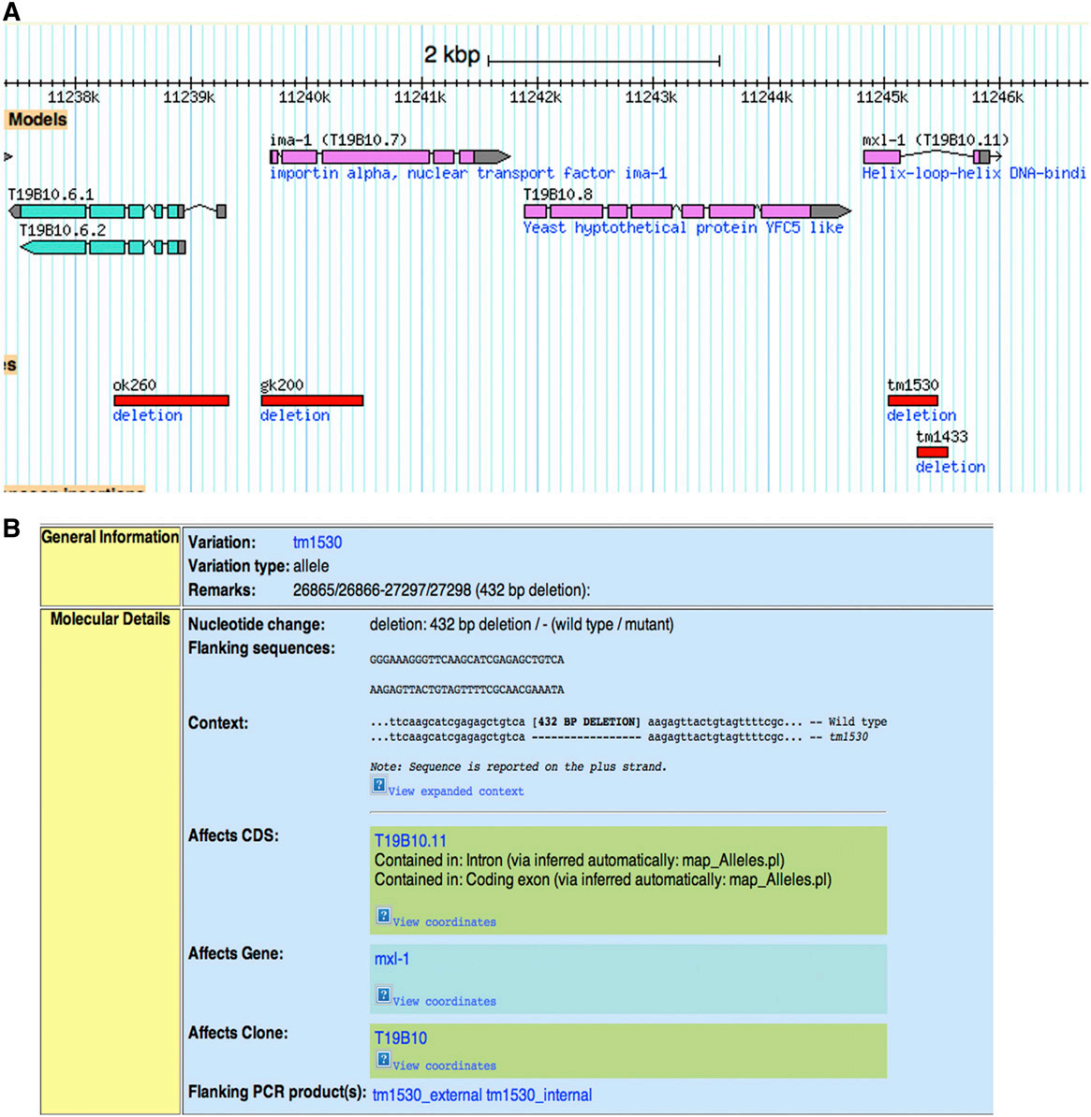
WormBase view of image and annotation for deletions. (A) Screenshot of four genes on chromosome V. Red bars denote deletions, and the length of the bar indicates size of the deletion. (B) The red bars in (A) are hot links to text describing the deletions in greater detail. Besides the details on deletion breakpoints, primers used to amplify the deletion region are listed. Depicted here is the link for deletion *tm1530*.

Once strains from the Vancouver and Oklahoma groups pass QC, they are shipped to the Caenorhabditis Genetics Center (CGC) in Minneapolis, Minnesota, for distribution (http://www.cbs.umn.edu/CGC/). All strains isolated in Tokyo are available by request from the Mitani Lab (http://shigen.lab.nig.ac.jp/c.elegans/index.jsp). All three laboratories submit data on each mutation, including sequence, flanking primers used for PCR and sequencing, mutagen employed, and strain background to WormBase (http://www.wormbase.org/). Figure 2 illustrates the type of detailed information relevant to each mutation available through WormBase.

### Identifying and stabilizing lethal mutants

The objective after PCR deletion screening and several rounds of sib selection is to obtain a homozygous strain bearing the deletion, but in a significant fraction of cases, it is not possible to derive a homozygous mutant strain. This usually indicates that the mutation resides in an essential gene. Often published RNAi studies give us forewarning that a gene is essential, although RNAi analysis and actual deletion phenotypes do not always agree (our unpublished results). Where feasible, we use chromosomal inversions or translocation chromosomes to balance recessive lethal deletion chromosomes. As over 80% of the genome is covered by such rearrangements, we can handle the bulk of lethal stains in this manner (Edgley *et al.* 1995). In the remaining cases, we use flanking double mutations or chromosomes with green fluorescent protein (GFP) insertions as local balancers. In this way, we were able to balance 1436 strains from the Oklahoma and Vancouver labs. We do little characterization of the lethal strains other than to determine arrest stage (embryonic or larval arrest, or adult sterility). It is important when working with deletion strains harboring mutations in so-called essential genes to remember that we have not ruled out the possibility the lethality is due to a tightly linked background mutation. It is the responsibility of the user to do a transgenic rescue experiment.

**Figure 3:**
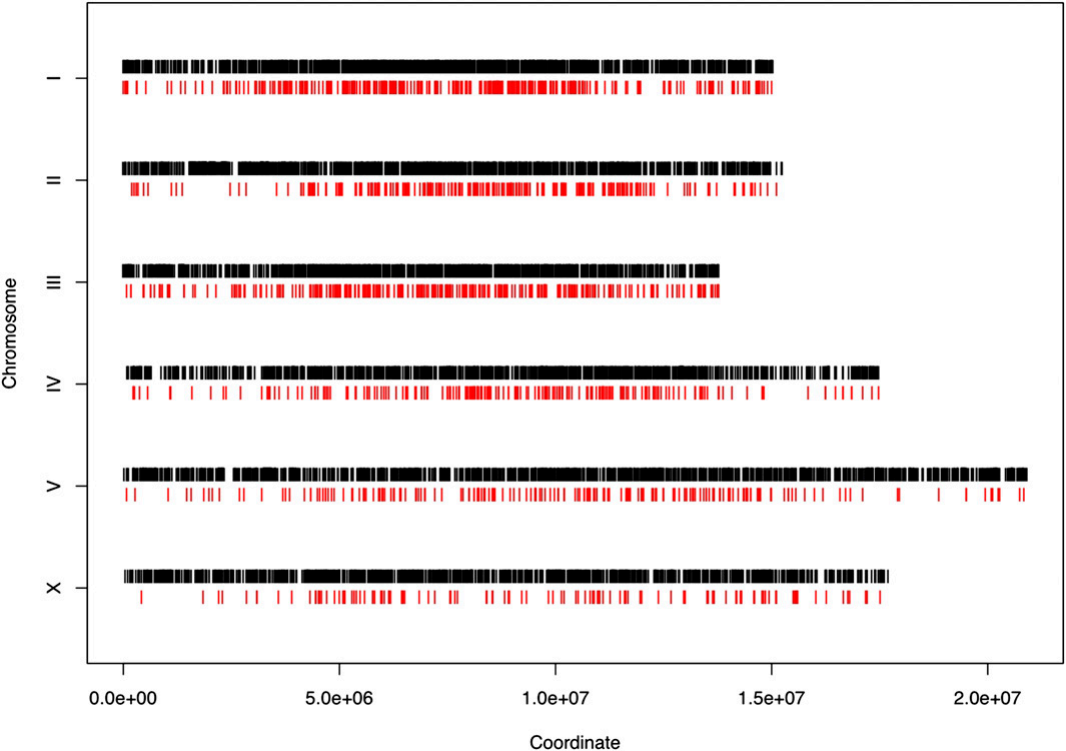
Comparison of distribution of all the mutations (black) and only the lethal mutations (red) throughout the whole genome. This figure is based on 6764 total genes and 1436 essential genes (WS220).

Out of the 6013 genes mutated in this study 1436, about 24%, can be mutated to a lethal phenotype. Of these 1436 genes, 946 are single-copy essential genes in the nematode. We did find at least one paralog for the remaining 490 genes. We were curious to determine the distribution along the chromosome of genes that can be mutated to a lethal phenotype. We found that both essential and non-essential genes are more or less evenly distributed along each chromosome (Figure 3). Regions of the chromosome arms where multi-gene families are enriched, particularly on chromosomes II and V, are the only regions with a somewhat reduced number of essential genes.

As *C. elegans* shares a large number of orthologous gene pairs with the yeast *Saccharomyces cerevisiae* (Chervitz *et al.* 1998), we were interested in determining whether they also share a significant overlap in essential genes. We used the program InParanoid (O’Brien *et al.* 2005) to identify 1905 pairs of orthologous genes between *C. elegans* and yeast. From the set of 1436 lethal genes in the nematode, only 413 have an ortholog in yeast. Of the 1193 essential genes in *Saccharomyces cerevisiae*, 678 have an ortholog in the nematode. The intersection of those 413 and 678 orthologous pairs yields 193 genes that are essential in both *C. elegans* and *Saccharomyces cerevisiae*. Although this is not a large number, some inferences can be made from this analysis. All 193 shared, essential genes between these two organisms are involved in core biological functions, such as DNA metabolism, protein synthesis, and energy production (see supporting information, Table S1 and GO annotation), a perhaps expected result when comparing the genome of a single-cell organism to a multicellular organism. It is also not too difficult to envisage a scenario where 485 genes essential in yeast are no longer essential in the worm, possibly through gene duplication and functional redundancy. As we only found paralogs in the nematode for 111 of these genes, gene duplication cannot be the answer in the majority of cases. More puzzling are the 220 orthologous genes not essential in yeast that are essential in the worm. Examination of the GO annotation for these orthologs did not distinguish them from the group of genes that are lethal in both organisms. When we examined the annotation for loss of function alleles in the Sacharomyces Genome Database (SGD; http://www.yeastgenome.org/), these yeast genes often had one or more of the following terms associated with the mutated state: vegetative growth decreased, colony sectoring, viability decreased, and/or slow growth. Perhaps what is marginal and on the edge of viability in yeast is terminal in the nematode.

### Targeting multi-gene families for knockouts

One significant difference between the genomes of *C. elegans* and *Saccharomyces cerevisiae* that presents a particular challenge to a biologist studying gene function is the expansion of shared gene families and the derivation of whole new gene families as one moves from a single-cell organism to the complexity of a multicellular organism. The degree of overlap in domains, the expansion of domain families, and the number of new domains in the nematode relative to yeast was first described by Chervitz *et al.* (1998) in their comparative analysis of the sequenced genomes of both model organisms. In addition to user requests for knockouts, we have endeavored to identify mutations in all members of certain gene families so the relative contribution of each gene to the function and phenotype of the animal can be determined.

Actin and actin-related proteins (*arp*) are examples of small gene families. While the Arp2/3 complex has a one-to-one ratio of genes between worms and yeast, actin itself is present as a single-copy gene in yeast, whereas there are five copies of the gene in the worm. There is a combination of similar and disparate tissue and temporal expression for these five actins (Krause *et al.* 1989; Avery 1993; MacQueen *et al.* 2005; Willis *et al.* 2006). While we have provided additional mutations to the existing actin mutant collection, our contribution has been more critical for the actin-related proteins, where we have provided the only alleles for three of the seven actin-related genes. This still leaves three members without mutations.

Other gene families with shared domains between yeast and nematodes have undergone a substantial expansion. Some examples of expanded gene families are as follows: protein kinases, which have expanded from 118 genes in yeast to 416 in the nematode; phosphatases, which have gone from 3 genes in yeast to 98 in the worm; helicases in yeast, while prominent at 17 copies, have ballooned to 85 genes in the nematode; PDZ-containing proteins, which have expanded from 2 genes in yeast to 64 in worms; Fibronectin type II domain–containing proteins have expanded from 2 genes in yeast to 47 in the nematode; LIM domain proteins, which have expanded from 3 genes in yeast to 30 in *C. elegans*; and MATH domain proteins, which have expanded from 1 gene in yeast to 86 in the nematode [all data from Chervitz *et al.* (1998), Hutter *et al.* (2009), GExplore (http://genome.sfu.ca/gexplore/), and WormBase (http://www.wormbase.org/)]. As can be seen in Table 1, we have obtained mutations in several genes for a diverse set of these expanded gene families, but we do not have mutations in all the members for any of the larger families.

**Table 1:** Mutations in multigene families in *C. elegans*

Gene Family*^a^*	Total Genes*^b^*	Number of Genes with Mutations	Percentage Complete
ABC transporters	58	57 (53)*^c^*	98%
Cadherin family	12	11 (9)	92%
Calmodulin-like EF hand	70	34 (25)	49%
Cytochrome p450	75	28 (28)	37%
Degenerin channels	30	24 (21)	80%
Epidermal growth factor domain	191	119 (98)	58%
Fibronectin type III domain	47	37 (35)	79%
GPCR rhodopsin	139	74 (73)	53%
GPCR orphan*^d^*	1,307	286 (281)	22%
Guanylate cyclase domain (30 are receptors)	38	36 (35)	95%
Helicases	85	53 (46)	62%
Heterotrimeric G proteins	22	21 (3)	95%
Innexins	25	25 (21)	100%
Kinases	416	380 (359)	91%
Ligand-gated ion channels	101	63 ((53)	62%
LIM domain	30	25 (24)	83%
LRR domain	56	26 (22)	46%
MATH domain*^e^*	86	70 (70)	81%
Metalloproteases	46	29 (28)	60%
microRNA (Mir)	207	92 (36)	44%
Neuropeptides	114	74 (73)	67%
Nuclear hormone receptors	275	250 (245)	91%
PAZ (Argonaute/ Dicer family)	26	25 (23)	96%
PDZ domain	64	41 (35)	63%
Phosphatases	98	28 (21)	29%
Potassium channels	72	40 (31)	56%
RRM (RNA recognition motif)	110	67 (56)	61%
Transcription factors*^f^*	941	866 (836)	90%
TRP (transient receptor potential channel)	21	21(19)	100%
Ubiquitin-like	25	14 (14)	56%
Ubiquitin conjugating enzyme E2	31	15 (13)	48%
Zinc finger proteins	831	655 (627)	79%

All data contributing to this table can be viewed in Table S2.

a Note that gene family members are not necessarily mutually exclusive. For example, many nuclear hormone receptors and zinc finger proteins are transcription factors.

b Data from GExplore and WB220.

c Data in brackets is the number of genes with a tm, ok, or gk allele (regardless of any other allele).

d Includes members of the *sra*, *srab*, *srb*, *srbc*, *srd*, *sre*, *srg*, *srh*, *sri*, *srj*, *srm*, *srr*, *srsx*, *srt*, *sru*, *srv*, *srw*, *srx*, *srxa*, and *srz* families. One hundred thirty-five (135) were found as *niDf* in wild-type strains.

e Only 11 have been detected by direct screening. The remaining 59 were found to be variable in wild-type strains.

f This number is a revised and updated list from Reece-Hoyes *et al.* (2005), Weirauch and Hughes (2011), and Hutter *et al.* (2009).

Mutations in all, or at least most, members of a gene family provide researchers with a powerful resource to study the functional importance of a particular gene in development and to determine its role in a variety of different tissues.

Innexins are an example of a gene family not found in yeast but only in multicellular organisms. These proteins are functionally analogous but not structurally homologous to connexins, vertebrate gap junction proteins. Innexins appear to perform the same function in many invertebrate species [reviewed in Phelan and Starich (2001)]. In *C. elegans*, there are 25 innexins, and between our efforts and those of the community, there are now mutations in all 25 members of the family (see Table 1). Our facilities provided mutations for 22 of these genes, and in 18 instances, we provided the canonical allele. Members of this gene family are expressed in different combinations, in different tissues, and at different times in development (Altun *et al.* 2009). Mutations in all 25 innexin genes provide the foundation to determine how these proteins contribute to all aspects of development. The cadherins are another family of proteins not found in yeast but that are critical for cell-cell adhesion in multicellular organisms (Pettitt 2005). In the nematode, there are 12 family members, and 11 have been successfully mutated. For 5 of these 12 genes, the only mutations are those provided by the Deletion Consortium.

Often a requesting laboratory asks for all members of a small gene family to be targeted. The transforming growth factor (TGF) beta protein family is a case in point. There are 5 members of the family, and although the community had already obtained mutations in 3 of the genes, we were able to provide mutations in the remaining 2, as well as additional alleles of the previously mutated 3. The nuclear hormone receptor (NHR) family with 275 genes is one of the larger gene families in the nematode. These genes have diverse roles in metabolism, homeostasis, and development, but only a few have so far been characterized in the worm [reviewed in Antebi (2006)]. We provided deletion mutations in 245 of the 250 mutated NHR genes (Table 1). With mutations in over 90% of these genes, there is an opportunity to determine the role of these proteins in the context of complex endocrine and other signaling networks. Our progress in obtaining mutations in several other gene families unique to metazoans is shown in Table 1.

### Genes expressed in the nervous system

Because there is a complete cell lineage and a complete wiring diagram of the nervous system (Sulston and Horvitz 1977; Sulston *et al.* 1983; White *et al.* 1976), *C. elegans* has emerged as an organism of choice for studies in neurobiology. These unprecedented cell-mapping resources mean that, in the worm, the origin of each neuron is known and all synaptic connections are mapped. With the addition of a sequenced genome, it was revealed that many molecular pathways are shared between the nematode and vertebrate nervous systems (Bargmann 1998). Animal movement, feeding, and behavior are all regulated through the nervous system, and this requires about one third of all somatic cells. In cooperation with the *C. elegans* community, we have strived to obtain mutations in genes and gene families contributing to nervous system function, with the expectation that this will facilitate efforts to describe in detail how neural networks control behavior in this organism.

A group of 30 genes encodes a class of sodium channels named degenerins. The best-studied members of this gene family are involved with touch or stretch sensation (Mano and Driscoll 1999). Within this family, there are 16 genes with mutations identified only by our group. The nematode also has voltage-activated calcium channels and other types of calcium channels, as well as a large and varied family of potassium channels. The community, with some support from our group, has identified mutations in most of the voltage-activated calcium channel genes, and we aided in identifying mutations in two calcium leak channels, *unc-77* (aka *nca-1*) and *nca-2*. Obtaining mutations in all 70-plus potassium channel genes has proven to be more elusive. The genes encoding potassium channels are divided into at least eight conserved families, and many have mammalian orthologs. These families fall into three groups: 2TM (transmembrane) potassium channels (3 genes), 4TM potassium channels (47 genes), and 6TM potassium channels (22 genes) [Salkoff *et al.* (2005); revised numbers from GExplore and WormBase]. There are now mutations in 40 of the 72 genes, but the *twk* 4TM family is still lacking mutations in half its members. The 6TM family is better covered, partially through our efforts, but primarily through the efforts of individual laboratories also using PCR deletion screening strategies [see, for example, Wei *et al.* (2002)]. Further selected examples of knockouts for genes expressed in the nervous system are described below and listed in Table 1.

The transient receptor potential (TRP) channel family is composed of a diverse set of proteins involved in mechanosensation and other responses to the environment (Goodman 2006). For this family, there are mutations in all 21 genes, and several of these only have a knockout allele provided by the Deletion Consortium. Neuropeptides are another group of proteins important in the nervous system for modulating behavior. There are 114 genes encoding neuropeptides, and these comprise three gene families: the insulin family of 40 genes; the FMRamide family of 30 genes; and the non-insulin and non-FMRamide family of 44 genes (Li and Kim 2008). The availability of mutations in at least 75 of these genes will allow a detailed investigation into the modulatory role of this important class of peptides in *C. elegans* behavior.

There are an estimated 101 ligand-gated ion channels in the nematode. These include excitatory receptors for acetylcholine and glutamate and inhibitory receptors for GABA and glutamate [Robertson and Thomas (2006); revised numbers from GExplore and WormBase]. The combined efforts of the community and our group have identified mutations in over 60% of these genes. It should be noted that targeted deletions for many of these genes are absolutely essential, as the knockout phenotype of most of these genes is very weak or requires specific assay conditions to detect. Mutations in these genes would not be detected in most forward mutation screens. This is true in general for most genes in the larger gene families, but it is an especially acute problem for the nervous system, where one is often dealing with very subtle phenotypes.

*C. elegans* has an inordinate fondness for 7TM G-protein–coupled receptors (GPCR). As pointed out by both Bargmann (1998) and Robertson and Thomas (2006), this class of protein accounts for 5–7% of all *C. elegans* genes. Two types of GPCR protein are described, those with clear homologs in other animals (139 genes), and “orphan” or worm-specific receptors, a group consisting of over a thousand genes in several subfamilies. For the group with clear homologs in other organisms, we have obtained mutations in about half of the genes (74 of 139). The number of functional genes within the orphan receptors is not clear as many have stop codon and missense mutations, but estimates are of about 1300 intact genes and more than 400 pseudogenes (Robertson and Thomas 2006; Hutter *et al.* 2009; GExplore). The estimate of 400 pseudogenes is based on examination only of the N2 Bristol strain, so many of these genes could be functional in other wild isolates. Our group has provided all but 5 of 286 lesions in the intact genes, and about half of these are due to *niDf* variants in wild-type populations (Table 1; Maydan *et al.* 2010).

### RNA metabolism, regulation, and miRNA

A very active area of research within the *C. elegans* community involves the maturation, regulation, and turnover of mRNA. Proteins with RNA recognition motif (RRM) domains often regulate message splicing and other aspects of mRNA function. There are over a hundred proteins containing RRM domains in *C. elegans*, and we as well as the community have identified mutations in over half of them (numbers from GExplore; Table 1).

The regulation of mRNA expression via micro RNAs (miRNA) complementary to the 3′UTR of messages is an active area of research with new examples being discovered frequently. The interaction of the *lin-4* and *lin-14* genes was the first example of this type of regulation [reviewed in Ambros (1997)]. Since then, hundreds of miRNA genes have been discovered in many species (Ambros 2004). The current estimate for the total number of miRNA genes in *C. elegans* is 207 [Kaufman and Miska 2010; MirBase (http://www.mirbase.org/cgi-bin/mirna_summary.pl?org=cel)], but it should be noted that not all of these genes are confirmed, as it is difficult to identify miRNA genes through bioinformatic approaches alone. Just over 40% of these genes have been deleted, and most do not display an overt phenotype (Miska *et al.* 2007). Although the Deletion Consortium has provided deletions for only a portion of these genes, our group developed the “poison primer” protocol used to obtain deletions in miRNA genes (Edgley *et al.* 2002). These genes are difficult to target due to their small size, and the poison primer method proved invaluable for detecting deletions in specific small regions.

When the Deletion Consortium was formed, its mission was to provide targeted mutations, which at the time was the only route to systematically examine loss-of-function phenotypes. The development of RNA interference (RNAi) as a convenient and rapid epigenetic method to generate mutant phenocopies in *C. elegans* is a popular and viable alternative to gene knockouts (Fire *et al.* 1998; Fraser *et al.* 2000; Gönczy *et al.* 2000; Ashrafi *et al.* 2003; Kamath *et al.* 2003; Barstead 2001). RNAi and deletions each serve a purpose, and often these approaches complement one another. Many laboratories perform RNAi screens in sensitized backgrounds, which usually require a null allele of a gene in a particular pathway of interest. Often the follow-up to an RNAi experiment is a request to a participating laboratory in the Deletion Consortium for a deletion allele. Determining the molecular details of RNAi itself require knocking out many of the genes involved, including Dicer and other Argonaute proteins, all members of the PAZ domain protein family [Table 1; for example, see Knight and Bass (2001)].

### Transcription factors and kinases

A major goal of our three laboratories is to obtain mutations for all 941 genes encoding transcription factors and the 416 genes encoding kinases. Our reason for targeting these two large gene families is that together they represent two major levels of developmental control within an organism. Although we have not completed either set, we are close in both cases, with mutations in 866 (90%) transcription factor genes and 380 (91%) kinase genes (Table 1). When combined with the current efforts of modENCODE (http://www.modencode.org/), the mutations in transcription factors should be especially valuable over the coming years to help dissect the transcriptional networks controlling development in this organism. Likewise, unraveling the complexity of intracellular signaling cascades will be greatly aided with mutations in all the kinases. The capacity for this type of analysis is unprecedented for any other metazoan.

### Summary

Deletion strains are an enduring community resource because worm stocks can be frozen and then thawed when needed. Public-domain generation of knockouts by dedicated projects (such as the Deletion Consortium) and availability of the stocks from central distribution nodes (the CGC or Tokyo) reduces unnecessary redundancy that could result if the targeted production of *C. elegans* mutants were left solely to individual investigators. Actual request and distribution statistics reveal the magnitude of wasteful duplication of effort that has been avoided. First, on average, the targets on our list have been requested by at least two investigators. In fact, we have received up to 10 requests for the same target. Second, strains from the CGC are shipped on average to three or four investigators.

The current state of our efforts provides a wide range of new research opportunities into fundamental questions in biology. A drawback for *C. elegans* researchers in the past has been the lack of tools to directly alter a specific locus. Our deletion mutation collection partially offsets this limitation. As well, several technical developments, including the use of *Drosophila mauritiana Mos1* transposons (Bessereau 2006), engineered zinc-finger containing DNA binding proteins (ZFN; Urnov *et al.* 2010), and transcription activator-like effector domain nucleases (TALEN; Boch 2011; Bogdanove and Voytas 2011; Li *et al.* 2011) will help to circumvent these limitations and are already changing the landscape for performing gene deletion and replacement experiments. The toolkit for *Mos1* manipulation for specific gene deletions and modifications is impressive and relies on transgene conversion of a site after *Mos1* excision has generated a double-strand DNA break. Variations on the theme include *Mos1* excision–induced transgene-instructed gene conversion (MosTIC) (Robert and Bessereau 2007); *Mos1*-mediated single-copy insertion (MosSCI) (Frøkjaer-Jensen *et al.* 2008); and *Mos1*-mediated deletion (MosDEL) (Frøkjaer-Jensen *et al.* 2010). TALENs offer many of the same features and do not require a resident nearby transposon. Successful gene targeting in the worm using transcription activator-like effector domain was recently reported (Wood *et al.* 2011). The addition of a toolkit to custom design and make TALENs will make this a popular method to generate deletions and gene modifications in several model systems (Cermak *et al.* 2011). In addition to these techniques, massively parallel short-read sequencing is becoming more widely adopted (Sarin *et al.* 2008; Flibotte *et al.* 2010). For an example of how this technique can be applied to obtain single base alterations and indels across a whole genome, see the Million Mutation project (http://genome.sfu.ca/mmp/about.html).

Over the next few years, the pace of obtaining identified mutations in genes will increase as these new approaches for obtaining and identifying mutations are applied to this organism. The combination of these diverse approaches in *C. elegans* should eventually lead to mutations in all genes. This knowledge will usher in a new age of metazoan genetics in which the contribution to any biological process can be assessed for all genes.

## Supplementary Material

Supporting Information

## APPENDIX

### PARTICIPANTS IN THE *C. ELEGANS* DELETION MUTANT CONSORTIUM

Barstead Laboratory, Molecular and Cell Biology, Oklahoma Medical Research Foundation, Oklahoma City, OK 73104.

Robert Barstead, Gary Moulder, Beth Cobb, Stephen Frazee, Diane Henthorn, Jeff Holmes, Daniela Jerebie, Martin Landsdale, Jamie Osborn, Cherilyn Pritchett, James Robertson, John Rummage, Ed Stokes, and Malani Vishwanathan.

Mitani Laboratory, Department of Physiology, Tokyo Women’s Medical University School of Medicine, Tokyo 162-8666, Japan.

Shohei Mitani, Keiko Gengyo-Ando, Osamu Funatsu, Sayaka Hori, Rieko Imae, Eriko Kage-Nakadai, Hiroyuki Kobuna, Etsuko Machiyama, Tomoko Motohashi, Muneyoshi Otori, Yuji Suehiro, and Sawako Yoshina.

Moerman Laboratory, Department of Zoology and Michael Smith Laboratories, University of British Columbia, Vancouver, BC, V6T 1Z3 Canada.

Donald Moerman, Mark Edgley, Ryan Adair, BJ Allan, Vinci Au, Iasha Chaudhry, Rene Cheung, Owen Dadivas, Simon Eng, Lisa Fernando, Angela Fisher, Stephane Flibotte, Erin Gilchrist, Allison Hay, Peter Huang, Rebecca Worsley Hunt, Christine Kwitkowski, Joanne Lau, Norris Lee, Lucy Liu, Adam Lorch, Candy Luck, Jason Maydan, Sheldon McKay, Angela Miller, Greg Mullen, Candice Navaroli, Sarah Neil, Rebecca Hunt-Newbury, Mikhaela Partridge, Jaryn Perkins, Anna Rankin, Greta Raymant, Nadereh Rezania, Alexandra Rogula, Bin Shen, Greg Stegeman, Angela Tardif, Jon Taylor, Mariana Veiga, Tina Wang, and Rick Zapf.
