# Supplementary material for: Large-Scale Screening for Targeted Knockouts in the Caenorhabditis elegans Genome
Source: G3 (Bethesda). 2012 Nov 1;2(11):1415–25. doi: 10.1534/g3.112.003830 (PMC3484672; doi:10.1534/g3.112.003830)
Supplement: Supporting Information [file supp_2_11_1415__index.html]

Supporting Information 

# Large-Scale Screening for Targeted Knockouts in the *Caenorhabditis elegans* Genome

## Supporting Information for the *C. elegans* Deletion Mutant Consortium, 2012

**Files in this Data Supplement:**

- Supporting Information - Tables S1 and S2 (PDF, 35 KB)
- Table S1 - Orthologous genes essential in yeast and nematodes (.xls, 57 KB)
- Table S2 - All data compiled from GExplore and WB220 (.xls, 603 KB)
