# Supplementary material for: Large-Scale Screening for Targeted Knockouts in the Caenorhabditis elegans Genome
Source: G3 (Bethesda). 2012 Nov 1;2(11):1415–25. doi: 10.1534/g3.112.003830 (PMC3484672; doi:10.1534/g3.112.003830)
Supplement: Supporting Information [file supp_2.11.1415_003830SI.pdf]

Tables S1 and S2

Available for download at http://www.g3journal.org/lookup/suppl/doi:10.1534/g3.112.003830/-/DC1.
